# Vertical variation of bacterial production and potential role in oxygen loss in the southern Bay of Bengal

**DOI:** 10.3389/fmicb.2023.1250575

**Published:** 2023-11-08

**Authors:** Wenqi Ye, Xiao Ma, Chenggang Liu, Ruijie Ye, W. N. C. Priyadarshani, Ruchi Jayathilake, Ashoka Weerakoon, Udeshika Wimalasiri, P. A. K. N. Dissanayake, Gayan Pathirana, R. G. A. Iroshanie, Yuanli Zhu, Zhongqiao Li, Bin Wang, Lu Shou, Lihua Ran, Feng Zhou, Jianfang Chen, Ping Du

**Affiliations:** ^1^Key Laboratory of Marine Ecosystem Dynamics, Second Institute of Oceanography, Ministry of Natural Resources (MNR), Hangzhou, China; ^2^State Key Laboratory of Satellite Ocean Environment Dynamics, Second Institute of Oceanography, MNR, Hangzhou, China; ^3^National Institute of Oceanography and Marine Sciences, National Aquatic Resources Research and Development Agency (NARA), Colombo, Sri Lanka; ^4^Department of Oceanography and Marine Geology, Faculty of Fisheries and Marine Sciences & Technology, University of Ruhuna, Matara, Sri Lanka

**Keywords:** Bay of Bengal, OMZ, bacterial production, diapycnal oxygen supply, bacterial oxygen consumption

## Abstract

Marine environments wherein long-term microbial oxygen consumption exceeds oxygen replenishment can be associated with oxygen minimum zones (OMZ). The Bay of Bengal OMZ (BOB-OMZ) is one of the most intense OMZs globally. To assess the contribution of bacterial oxygen consumption to oxygen loss in BOB-OMZ, we measured bacterial production (BP), temperature, salinity, and dissolved oxygen (DO) in the whole water column. We then compared the estimated bacterial oxygen demand (BOD) with diapycnal oxygen supply (DOS) at depths of 50–200 m in the southern BOB in January 2020. The average BP was 3.53 ± 3.15 μmol C m^−3^ h^−1^ in the upper 200 m of four stations, which was lower than those reported in other tropical waters. The vertical distribution of BP differed between the open ocean and nearshore areas. In the open ocean, temperature and DO were the most important predictors for BP in the whole water column. In the nearshore areas, when DO increased sharply from the suboxic state, extremely high BP occurred at 200 m. The average estimated BOD/DOS could reach up to 153% at depths of 50–200 m, indicating advection and anticyclonic eddies probably are important DO replenishment pathways in the BOB.

## 1. Introduction

The oxygen minimum zone (OMZ), first proposed by Cline and Richards (1972), is defined as dissolved oxygen (DO)-deficient mesopelagic waters. Widespread and intensive OMZ has an impact on the biogeochemical cycles. Anammox and denitrification occur in hypoxic conditions, especially under conditions of DO <5 μmol kg^−1^ (Gruber, 2011; Bristow et al., 2017). In these processes, on the one hand, the production of greenhouse gas N_2_O contributes to the global warming, and on the other hand, the loss of nitrogen limits the primary production in the ocean (Falkowski, 1997; Codispoti et al., 2001; Paulmier and Ruiz-Pino, 2009).

Along with the Arabian Sea OMZ (AS-OMZ), the Bay of Bengal OMZ (BOB-OMZ) is one of the most intense OMZs globally, the combination of which account for 59% of the total global OMZ area (defined as areas where DO <0.5 mL L^−1^; Helly and Levin, 2004). The formation of BOB-OMZ is strongly related to stratification stability and oxygen consumption in the upper water. Heavy precipitation (~0.8–2 m yr.^−1^), large terrigenous freshwater input (~1.6 × 10^12^ m^3^ yr.^−1^), and weak ventilation have led to stable stratification and weak convective mixing, which in turn have limited the replenishment of surface DO to the middle and deep water (Subramanian, 1993; Han and Webster, 2002; Prasanna Kumar et al., 2002). Additionally, the nutrient input by terrigenous runoff promotes the growth of phytoplankton and generates continuous aerobic degradation in the process of downward subsidence (Sarma, 2002), which accelerates oxygen consumption in the upper water. In addition, the higher nutrients and organic matter could fuel microbial activities, and the enhanced respiration intensifies oxygen deficiencies in the upper layer and in the benthic mixed layer of the Bengal Fan (Broecker et al., 1980; Toyoda et al., 2023). The deep Indian Ocean water shows a very definite reduction in oxygen and increases in silicate, nitrate, and phosphate toward the north along any given north/south section (Broecker et al., 1980).

In contrast to other OMZs, the BOB-OMZ has no significant denitrification, although the DO level could reach the condition for denitrification (Naqvi et al., 1994; Rao et al., 1994). Naqvi et al. (1994) attributed this to low primary production, rapid sedimentation of organic matter, and low bacterial respiration rates, which make the BOB-OMZ less severe than the AS-OMZ and the Eastern Tropical South Pacific OMZ (ETSP-OMZ). However, the oxygen level in the core of the BOB-OMZ was much lower than that previously reported (Bristow et al., 2017). Bristow et al. (2017) hypothesized that a slight increase in primary production owing to enhanced nutrients input might intensify hypoxia and trigger anammox and denitrification. In contrast, Sridevi and Sarma (2020) argued that the sharp reinforcement of the BOB-OMZ was a random and short-term state, the DO in the OMZ would be balanced by physical forcing, including stratification, and the occurrence of cyclonic and anticyclonic eddies. In summary, both physical and biological activities sustain the BOB-OMZ. However, how biological oxygen consumption and physical forcing balance the DO in the OMZ remains unclear.

Rapid depletion of DO occurs mainly in the upper waters, especially the mixed layer depth to about 100 m, and the OMZ core (DO concentration lower than 20 μmol kg^−1^) often appears about 100 m (Paulmier and Ruiz-Pino, 2009). Heterotrophic bacterioplankton is the main contributor to rapid oxygen depletion in the upper ocean and thus maintains the OMZ (Thamdrup et al., 2012; Kalvelage et al., 2015). However, only a few reports have quantified the contribution of bacterial oxygen consumption to OMZ. Loginova et al. (2019) and Maßmig et al. (2020) showed that bacterial decomposition activities comprised up to 38 and 62% of diapycnal oxygen supply (DOS) in the upper oxycline during austral summer and austral winter in the ETSP, respectively. In the following study we will define bacterial contributions to oxygen supply as the ratio of bacterial oxygen demand (BOD) to DOS. Maßmig et al. (2020) estimated BOD using the following formula: BOD = (bacterial carbon demand [BCD] − bacterial production [BP]) / respiratory quotient. Bacterial production is the rate of biomass synthesized by bacteria using organic precursors (Ducklow, 2000). Bacterial production above the hypoxic layer in the BOB has been measured previously (Fernandes et al., 2008; Ramaiah et al., 2010); however, BP distribution across the BOB-OMZ remains unclear. Previous studies have indicated that bacterial respiratory processes are stimulated by a high input of labile organic matter (Chang et al., 2010; Kalvelage et al., 2013). Chlorophyll-*a* concentration and labile organic matter in the BOB are generally lower than those in the ETSP (Loginova et al., 2019), suggesting that BP and BOD in the BOB may also be lower. Compared to ETSP, stable stratification in the BOB may imply that DOS is also lower. However, it is difficult to predict whether the ratio of BOD to DOS in the BOB is higher or lower than that in the ETSP.

Therefore, we studied the distribution pattern of BP and its relationship with environmental factors in the whole water column in the southern BOB in January 2020. According to the methods of Maßmig et al. (2020) used in the ETSP, we calculated the BOD and DOS to reveal the contribution of bacterial activities to the persistence of the BOB-OMZ. This is the first report demonstrated the vertical distribution of bacterial production across the BOB-OMZ and quantified the bacterial contribution to the diapycnal oxygen loss in the southern BOB.

## 2. Materials and methods

### 2.1. Study area

**Figure 1:**
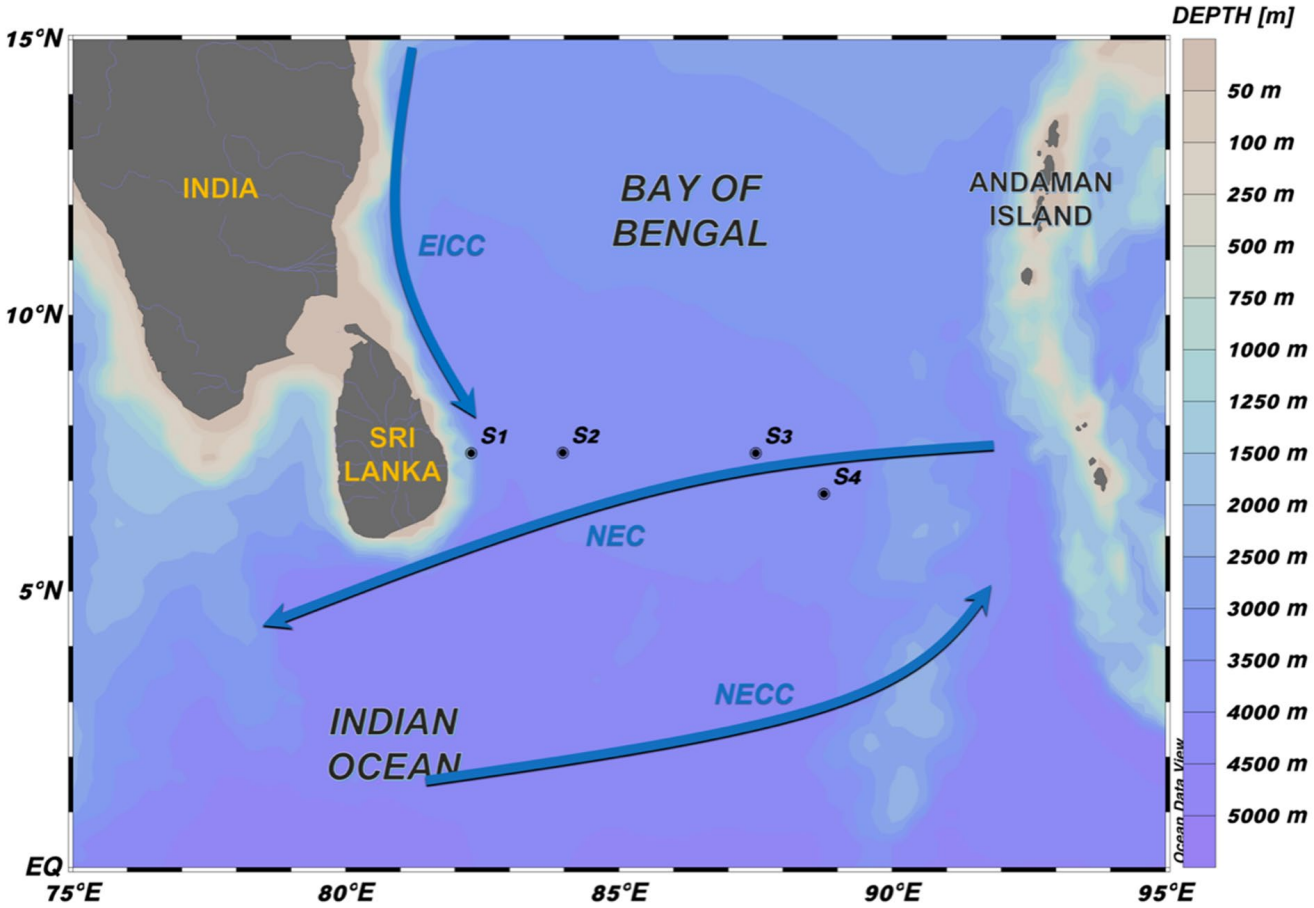
Study area. Stations S1–S4 were sampled in the southern BOB during January 8–31, 2020. S1 is in the nearshore areas, and S3–S4 are in the open ocean. Winter currents have been mentioned in previous studies (Hacker et al., 1998; Wijesekera et al., 2015).

The study area was located at the southern BOB, east of Sri Lanka, which serves as an important region for water exchange between the inside and outside of the bay (Figure 1; Schott et al., 1994). This region is characterized by monsoon that is the primary factor for seasonal circulation of the BOB. The southwest monsoon prevails in summer (May–October), whereas the northeast monsoon prevails in winter (November–February). During winter, the north Indian Ocean circulation is constituted of the westward North Equatorial Current (NEC), southward Somali Current (in the southwestern Arabian Sea), and North Equatorial Countercurrent (NECC; Potemra et al., 1991). In addition, the East India Coastal Current (EICC), one of the most important currents in the BOB, flows along the western boundary of the BOB. During October to December, the EICC moves southward along the entire eastern coast of India, whereas it moves northward during February to August; the direction changes in January and September (Legeckis, 1987; Shetye et al., 1993; Schott et al., 1994; Shankar et al., 1996).

According to previous studies, the sea surface temperature (SST, 29–30°C) is warm throughout the year in the southern BOB (south of 13°N; Narvekar and Kumar, 2006). Sea surface salinity (SSS) is higher (34.0–34.5) during the southwest monsoon than during the northeast monsoon (33.5–34; Jensen et al., 2016). Chlorophyll-*a* stocks were higher during the winter monsoon (~1,040 mg C m^−2^) and spring inter-monsoon (~1,000 mg C m^−2^) and lower during fall inter-monsoon (~836 mg C m^−2^) and summer monsoon (~603 mg C m^−2^). The seasonal variation in chlorophyll-*a* stocks is consistent with that in primary production (winter monsoon: ~304 mg C m^−2^d^−1^, spring inter-monsoon: ~328 mg C m^−2^d^−1^, fall inter-monsoon: ~294 mg C m^−2^d^−1^, summer monsoon: ~235 mg C m^−2^d^−1^; Ramaiah et al., 2010).

### 2.2. Field collection

Water samples from the four stations in the southern BOB were taken during the cruises “Joint Advanced Marine and Ecological Studies in the Bay of Bengal and the Eastern Equatorial Indian Ocean” cruises on the *R/V Xiangyanghong 06* from January 8, 2020 to January 31, 2020 (Figure 1). Temperature, salinity, DO, density, and depth were measured using a Sea-Bird SBE 9-plus CTD system (Sea-Bird Electronics Inc., United States). Water was sampled using 24 Niskin bottles (12 L) on a general oceanic rosette system. The DO measurement at each depth using an SBE 43 oxygen sensor was calibrated using Winkler titration (Winkler, 1888). Linear regressions between sensor and sample DO concentration data were analyzed as follows: DO_LAB_ = 0.98DO_SBE_ **–** 0.02 (*R*^2^ = 0.96, *n* = 20), where DO_SBE_ is the DO concentration recorded by the SBE 43 sensor, and DO_LAB_ is the DO concentration measured by the Winkler titration method.

### 2.3. Chlorophyll-*a* and DOC

At each station, chlorophyll-*a* measurements were sampled at seven layers between 2 m and 200 m (Supplementary Table S1). Chlorophyll-*a* concentration was measured by a fluorescence extraction method (Parsons, 2013). Chlorophyll-*a* concentration was analyzed fluorometrically (Turner Designs, United States, 10-AU-005-CE) by filtering 500 mL water samples from each depth using a GF/F filter (0.7 μm, Whatman) and extracting overnight in 8 mL 90% acetone at 4°C.

At S3 and S4, DOC measurements were sampled at seven layers between 2 m and 500 m (Supplementary Table S1). Samples (30 mL) in duplicate were filtered through pre-combusted 0.7 μm GF/F filters (Whatman). Filtrate was transferred into acid-rinsed and pre-combusted brown glass bottles and stored in a freezer (−20°C) for DOC measurements. The DOC concentration was measured by a high-temperature catalytic oxidation method (Cauwet, 1999). The DOC concentration was measured using the non-purgeable organic carbon mode of a Shimadzu TOC-L analyzer (Japan). With HCl and oxygen purging, inorganic carbon was removed. Then, the sample was injected into a platinum catalyst at a high temperature (680–700°C), and DOC that was oxidized to CO_2_ was measured using a non-dispersive infrared detector. The DOC concentrations were computed according to the manufacturer’s protocol. Potassium hydrogen phthalate standard solutions were used to establish a standard curve. Accuracy of the DOC concentration was checked using a 1 mg L^−1^ standard solution (diluted from 1,000 mg L^−1^ DOC standard). Each sample was measured at least twice until the variation coefficient reached less than 2%.

### 2.4. Bacterial abundance and production

At each station, bacterial abundance (BA) was sampled at seven or eight layers between 2 m and 200 m (Supplementary Table S1). Water samples (3.6 mL) were collected and fixed with paraformaldehyde at a final concentration of 1%. The samples were stored in liquid nitrogen and transported to the laboratory. Before analysis, the samples (1 mL) were thawed and stained with SYBR Green I (Molecular Probes) at a final concentration of 10^−4^ of the stock solution (10000×) supplied by the manufacturer (Lebaron et al., 1998). Total bacterial numbers were counted using a BD FACSCalibur flow cytometer (BD, United States; Marie et al., 1997).

Bacterial production was estimated from the rate of protein synthesis, as measured by the incorporation of [^3^H]-leucine (Kirchman et al., 1985; Simon and Azam, 1989). At each station, BP measurements were sampled at 10 or 11 layers between 2 m and 3,800 m (Supplementary Table S1) in replicates. Five centrifuge tubes of water samples were collected from each layer. Water was collected following a protocol for DO measurements using the Winkler titration method. Excess seawater was quickly removed using a pipette, retaining 20 mL before operation. Two of the tubes were sterilized with 2 mL of 50% trichloroacetic acid (TCA) solution, to be used as blanks. Each tube was slowly added with [^3^H]-leucine reagent (1 mCi mL^−1^, specific activity = 60.0 Ci mmol^−1^, at a final concentration of 20 nmol L^−1^, PerkinElmer) along the wall to avoid bubbles. The cap was tightened and sealed, and the tube was shaken gently. Five centrifuge tubes of water samples were incubated at *in-situ* temperature in the dark temperature-controlled (−20–55°C) car refrigerator within half an hour after sampling. After 2 h of incubation, the temperature deviation is within 0.5°C, 2 mL of 50% TCA was added to each of the three parallel tubes to terminate the incubation. Then, the water sample was filtered through a 0.22-μm mixed cellulose filter. After filtration, the filter was rinsed with 5% TCA solution and 80% ethanol and drained. The TCA solution and ethanol were stored in an ice bath in advance. The filter was preserved in a scintillation bottle at −20°C. Ethyl acetate (0.5 mL) was added to dissolve the filter completely. The samples were radio-assayed in a ALOKA LSC-8000 liquid scintillation analyzer (Hitachi, Japan) using Ultima Gold (Packard) scintillation cocktail as the fluor. Bacterial production was calculated using the following formula (Eq. 1):


(1) $$BP=1.55\times 10^{6}\times Ri$$

where Ri (mmol Leu L^−1^ h^−1^) is the leucine absorption rate, BP (μg C L^−1^ h^−1^) is the bacterial production. As the conversion factor has not been measured in BOB and the influence of hypoxia on the conversion factor has not yet been systematically examined, thus, the conservative theoretical conversion factor of 1.55 × 10^6^ μg C mmol^−1^ Leu (Simon and Azam, 1989) was used for convenience of comparison with past records. Samples with a standard deviation >30% between replicates were excluded.

### 2.5. Calculations of bacterial oxygen demand and diapycnal oxygen supply

In this study, the bacterial contribution to the oxygen loss in BOB-OMZ was explained by comparing the BOD and DOS. Here, we calculated the BOD and DOS from the mixed layer depth (MLD; ~50 m) to 200 m at S3. Bacterial oxygen demand is the rate of oxygen consumption by bacterial respiration. Diapycnal oxygen supply is the negative vertical divergence of oxygen, which is the amount of oxygen that is lost per unit of time in a specific depth interval of the water column owing to organism utilization (Loginova et al., 2019).

Bacterial oxygen demand (BOD, μmol O_2_ m^−3^ h^−1^) was calculated as the difference between bacterial carbon demand (BCD, μmol C m^−3^ h^−1^) and BP (μmol C m^−3^ h^−1^) by applying a respiratory quotient (RQ) of 1 (Del Giorgio and Cole, 1998) as Eq. 2.


(2) $$\mathrm{BOD}=\left(BCD-BP\right)/RQ$$

The BCD (μmol C m^−3^ h^−1^) was calculated assuming that BGE follows the established temperature dependence (BGE = 0.374[±0.04] – 0.0104[±0.002]T, °C), where T is the *in-situ* temperature (Rivkin and Legendre, 2001). Thus, the BGE was between 10 and 25% in the depth range of 50–200 m at S3 based on the *in-situ* temperature of 13–26°C. The estimated BGE is used to calculate BCD from measured BP as Eq. 3 shown below.


(3) $$BCD=\frac{BP}{BGE}$$

Oxygen loss rates were calculated from the changes in diapycnal fluxes with depth, that is, the vertical divergence of DO. The oxygen profile (station S3) below the MLD (~50 m) was used to calculate the gradient of DO ($\nabla C_{\mathrm{DO}}$, μmol m^−4^).

The diapycnal flux of oxygen (Φ_DO_, μmol m^−2^ h^−1^) was estimated using Eq. 4 as follows:


(4) $$\Phi_{\mathrm{DO}}=-K_{\rho }\nabla C_{\mathrm{DO}}$$

The diapycnal diffusivity (*K_ρ_*, m^2^ s^−1^) was assumed to be constant (4.82 × 10^−5^ m^2^ s^−1^), which was the mean value between 50 m and 140 m obtained from turbulence measurements using a free-falling microstructure probe (see Text S1 for the detailed calculation of *K_ρ_* and error).

The mean diapycnal supply of oxygen (DOS, $-\bar{\nabla \Phi_{\mathrm{DO}}}$, μmol m^−3^ h^−1^) is defined as the negative vertical divergence of oxygen (Loginova et al., 2019), which was calculated from the mean diapycnal flux profile according to Eq. 5 below:


(5) $$-\bar{\nabla \Phi_{\mathrm{DO}}}=-\frac{\partial }{\partial_{z}}\bar{\Phi_{\mathrm{DO}}}$$

### 2.6. Statistical analyzes

The five water layers were divided according to the DO concentration (Gruber, 2011; Maßmig et al., 2020). Specifically, the water layers with DO >60 μmol O_2_ kg^−1^ was defined as “oxic,” whereas that with DO ≤60 μmol O_2_ kg^−1^ was defined as “OMZ.” The “core” of OMZ was delimited by DO ≤20 μmol O_2_ kg^−1^. The oxycline included the “upper OMZ” and “lower OMZ,” with DO ranging between 20 and 60 μmol O_2_ kg^−1^, respectively. We defined “suboxic” as a DO of ≤5 μmol O_2_ kg^−1^. The MLD was defined as temperature deviating ≤0.2°C from the maximum.

Data were plotted using Ocean Data View (Version 5.3.0) and Origin Pro 2021. Statistical significances of BP between different DO sections were tested using Kruskal–Wallis test in SPSS (Version 20.0). Spearman correlation analysis (two-tailed) of BP and cell-specific BP with environmental factors was performed using SPSS 20.0. All dependent variables were investigated using multiple linear regression (stepwise procedure; variables were included in the analysis when *p* < 0.05 and were excluded from the analysis when *p* > 0.10) to determine the possible effect of the independent variables in SPSS (Version 20.0). All linear regression analysis modes have excluded collinearity.

## 3. Results

### 3.1. Vertical profiles of physical and biochemical variables

**Figure 2:**
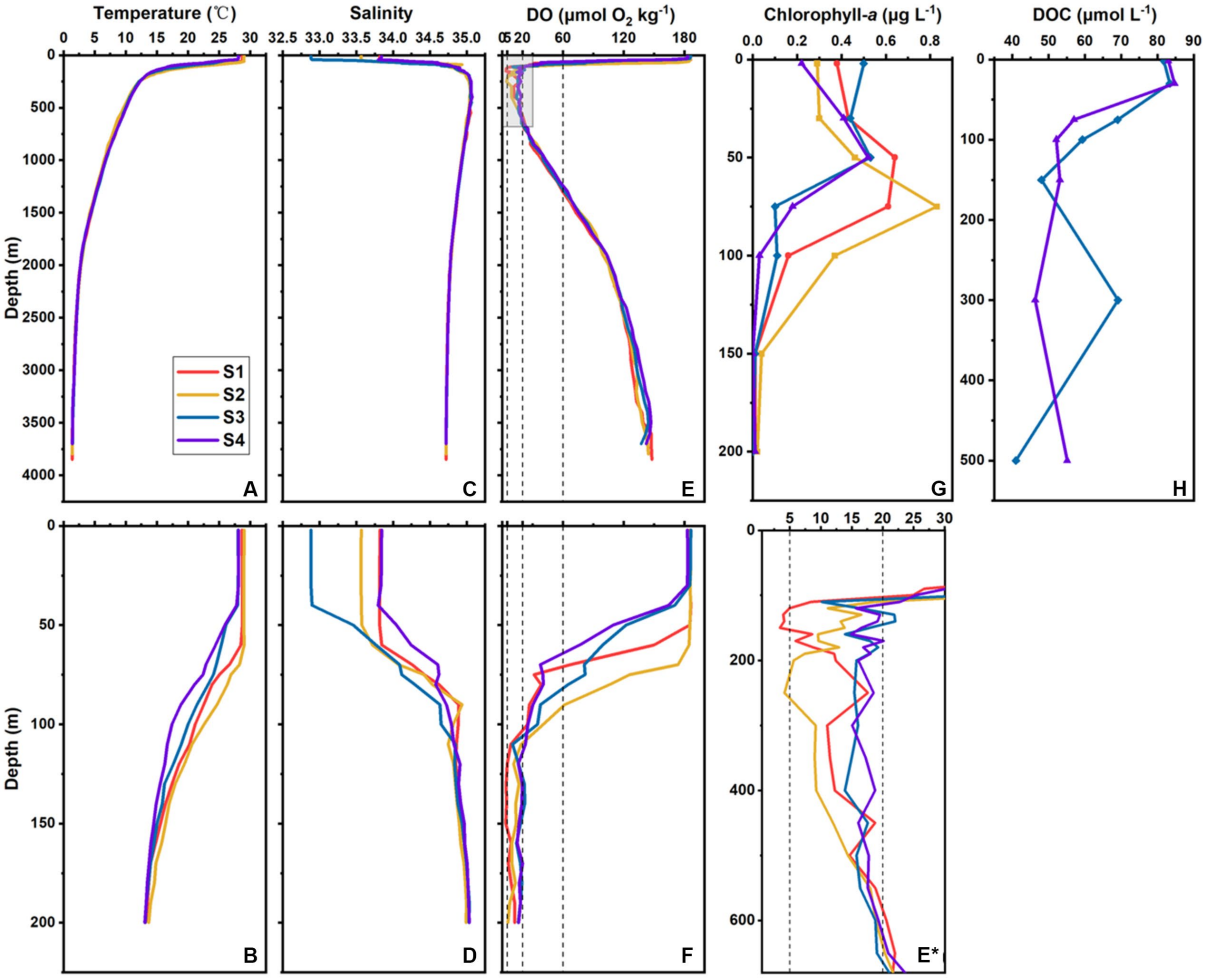
Vertical profiles of the environmental factors at S1–S4. Temperature (°C) in the whole water column **(A)** and upper 200 m **(B)**, salinity in the whole water column **(C)** and upper 200 m **(D)**, DO (μmol O_2_ kg^−1^) in the whole water column **(E)** and upper 200 m **(F)**, chlorophyll-*a* (μg L^−1^) in the upper 200 m **(G)**, and DOC (μmol L^−1^) in the upper 500 m **(H)** at different stations in the southern BOB. (E^*^) is the enlarged view of the gray box in **(E)**. Dotted lines in **(E)**, (E^*^), and **(F)** marked 5, 20, and 60 μmol O_2_ kg^−1^ DO, respectively.

The bottom depths of the four sampled stations were all ~4,000 m. The SST ranged from 28.1°C to 29.0°C and averaged 28.5°C ± 0.5°C. The SSS ranged 32.9–33.8 and averaged 33.5 ± 0.4. Obvious vertical stratifications of temperature and salinity were identified (Figures 2A–D). The MLD occurred at ~60 m at S1 and S2 and at ~40 m at S3 and S4. For all stations, the temperature decreased steeply in the upper 200 m and continued to decrease until approximately 2°C below 2,000 m (Figures 2A,B). The salinity at the four stations increased up to ~35 in the upper 250 m and decreased to ~34.7 below 2,000 m (Figures 2C,D).

The surface DO ranged from 183 to 186 μmol O_2_ kg^−1^ and averaged 185 ± 2 μmol O_2_ kg^−1^. The OMZ generally occurred at a depth of ~70–1,300 m. The OMZ core ranged from ~100 m to 600 m. Although the trends in oxygen concentration variation with depth among all stations were similar, their intensities were quite different. The most intense OMZ occurred at S1, which also had the largest suboxic range (120–150 m), whereas the suboxic layer of S2 occurred at ~250 m. Conversely, S3 and S4 were not suboxic (Figures 2E,E*,F).

In the upper 200 m, chlorophyll-*a* concentration ranged from below the detection limit to 0.83 μg L^−1^ and averaged 0.27 ± 0.24 μg L^−1^. In the surface (2 m depth, the same below), it ranged 0.22–0.50 μg L^−1^ and averaged 0.35 ± 0.12 μg L^−1^. The chlorophyll-*a* values of S1 and S3 were higher than those of S2 and S4 in the upper 30 m; those of S1 and S2 were higher than those of S3 and S4 below 30 m (Figure 2G). The deep chlorophyll maximum (DCM) depth of S2 was 75 m; for the other three stations, it was 50 m. For all stations below 150 m, the chlorophyll-*a* concentration was generally below the detection limit (Figure 2G).

The DOC concentration ranged from 41 to 85 μmol L^−1^ and averaged 63 ± 14 μmol L^−1^ in the upper 500 m water column at S3 and S4. At both S3 and S4, DOC reached its maximum value at 30 m (Figure 2H).

### 3.2. Bacterial production and cell-specific bacterial production

Bacterial production in the whole water column ranged from 0.05 to 12.29 μmol C m^−3^ h^−1^ in the four stations. In the upper 200 m, it ranged from 0.18 to 12.29 μmol C m^−3^ h^−1^ and averaged 3.53 ± 3.15 μmol C m^−3^ h^−1^ in the four stations. In the surface, it ranged from 2.07 to 6.38 μmol C m^−3^ h^−1^ and averaged 3.63 ± 2.11 μmol C m^−3^ h^−1^.

**Figure 3:**
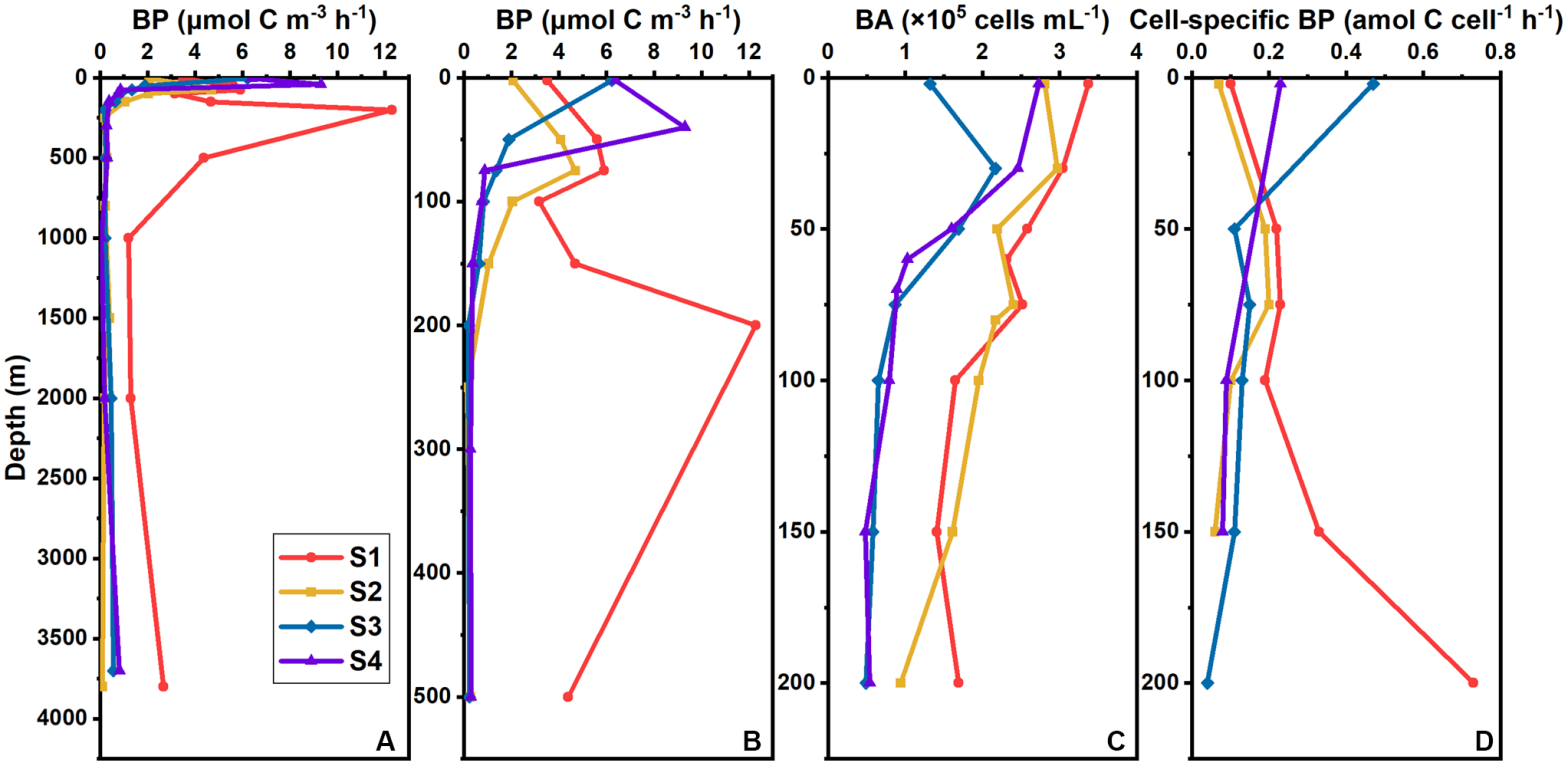
Vertical distribution of BP (μmol C m^−3^ h^−1^) in the whole water column **(A)** and upper 500 m **(B)**, vertical distribution of BA (×10^5^ cells mL^−1^) in the upper 200 m **(C)**, and vertical distribution of cell-specific BP (amol C cell^−1^ h^−1^) in the upper 200 m **(D)** at different stations in the southern BOB.

The average BP in the whole water column of S1 (5.85 ± 3.34 μmol C m^−3^ h^−1^) was much higher than that of the other stations (1.51 ± 2.26 μmol C m^−3^ h^−1^). The vertical variation in BP differed between S1 and those three stations (Figures 3A,B). At S1, BP varied drastically over the upper 500 m, and the maximum BP appeared at 200 m (Figures 3A,B), with an extremely high value of 12.29 μmol C m^−3^ h^−1^; it was much lower below 1,000 m and varied slightly (Figure 3A). There were no significant differences in the vertical variation patterns among S2, S3, and S4 (Figures 3A,B). For those three stations, BP was higher over the upper 200 m, but decreased rapidly and varied slightly below 200 m. The maximum BPs at S2, S3, and S4 occurred at 75 m, the surface, and 40 m, respectively.

In the upper 200 m, the BA ranged from 0.48 × 10^5^ to 3.37 × 10^5^ cells mL^−1^ and averaged (1.74 ± 0.86) × 10^5^ cells mL^−1^ for all stations. Bacterial abundance at S1 and S2 was higher than that at S3 and S4 (Figure 3C). The highest BA was observed at surface at S1 and S4 and at 30 m at S2 and S3. Bacterial abundance then decreased with depth, except for that at S1, where the value at 200 m was higher than those at 100 m and 150 m (Figure 3C).

We normalized BP to the cell abundance in the upper 200 m (Figure 3D). The cell-specific bacterial production (cell-specific BP) ranged from 0.04 to 0.73 amol C cell^−1^ h^−1^ and averaged 0.19 ± 0.16 amol C cell^−1^ h^−1^ for the four stations. The vertical variation of cell-specific BP was similar to that of BP. At S1, cell-specific BP increased with depth, reaching the maximum at 200 m. For the other three stations, the average cell-specific BP (0.15 ± 0.11 amol C cell^−1^ h^−1^) was lower than that at S1 (0.30 ± 0.22 amol C cell^−1^ h^−1^), with the maximum occurring at 75 m at S2, but at surface at S3 and S4.

We compared the BP and cell-specific BP in different oxygen concentrations (Table 1). At S1, both the average BP and cell-specific BP in the OMZ, especially in the core of OMZ, were higher than that in the non-OMZ, albeit without significant difference (*p* > 0.05). However, the results were different in the other three stations. At S2, S3, and S4, both the average BP and cell-specific BP in non-OMZ were significantly higher than those in OMZ (*p <* 0.05), and those in the oxycline were slightly higher than those in the core of OMZ, albeit without significant difference (*p* > 0.05).

**Table 1:** Comparison of BP and cell-specific BP in different stations and layers.

Station		Core-OMZ (DO ≤20 μmol O_2_ kg^−1^)	OMZ (DO ≤60 μmol O_2_ kg^−1^)	Oxycline (20 μmol O_2_ kg^−1^ ≤ DO ≤60 μmol O_2_ kg^−1^)	Non-OMZ (DO >60 μmol O_2_ kg^−1^)
S1	BP	7.12 ± 4.49 (*n* = 3)	5.27 ± 3.79 (*n* = 6)	3.42 ± 2.35 (*n* = 3)	3.27 ± 1.80 (*n* = 4)
	Cell-specific BP	0.53 ± 0.28 (*n* = 2)	0.37 ± 0.24 (*n* = 4)	0.21 ± 0.03 (*n* = 2)	0.16 ± 0.08 (*n* = 2)
S2, S3, S4	BP	0.37 ± 0.28 (*n* = 9)	**0.52 ± 0.50** (*n* = 16)	0.70 ± 0.68 (*n* = 7)	**2.57 ± 2.88** (*n* = 15)
	Cell-specific BP	0.07 ± 0.03 (*n* = 4)	**0.09 ± 0.03** (*n* = 7)	0.11 ± 0.02 (*n* = 3)	**0.20 ± 0.13** (*n* = 7)

*n* is the number of samples. Average BP (μmol C m^−3^ h^−1^) in the whole water column and cell-specific BP (amol C cell^−1^ h^−1^) in the upper 200 m water column were compared among the different layers in the open ocean (S2, S3, and S4) and nearshore area (S1). Bold font indicates the difference is significant, *p* < 0.05.

### 3.3. Relationship between environmental factors and BP

Owing to the significant difference in BP between S1 and the other three stations, correlation analysis with the environmental factors was conducted for the two regions separately (Table 2). There was no significant correlation between BP and any environmental factors (temperature, salinity, and DO; *p* > 0.05) at S1. Similarly, there was no significant correlation between cell-specific BP and any environmental factors (temperature, salinity, DO, BA, and chlorophyll-*a*; *p* > 0.05) in the upper 200 m of the water column at S1. However, BA showed a significant positive correlation with DO in the upper 200 m at S1.

**Table 2:** Spearman correlation coefficients of BP and cell-specific BP with environmental factors.

		Temperature	Salinity	DO	BA	Chlorophyll-*a*
Whole Water column	BP (S1)	0.588 *n* = 10	−0.055 *n* = 10	−0.370 *n* = 10	–	–
	BP (S2, S3, S4)	0.791^**^ n = 31	−0.701^**^ n = 31	0.498^**^ n = 31	–	–
Upper 200 m Water column	BP (S1)	−0.257 *n* = 6	0.257 *n* = 6	−0.200 *n* = 6	−0.086 *n* = 6	0.000 *n* = 6
	BA (S1)	0.771 *n* = 6	−0.771 *n* = 6	0.943^**^ n = 6		0.725 *n* = 6
	Cell-specific BP (S1)	−0.714 *n* = 6	0.714 *n* = 6	−0.771 *n* = 6	–	−0.522 *n* = 6
	BP (S2, S3, S4)	0.903^**^ n = 14	−0.793^**^ n = 14	0.868^**^ n = 14	0.859^**^ n = 14	0.836^**^ n = 14
	BA (S2, S3, S4)	0.868^**^ n = 14	−0.657^*^ n = 14	0.714^**^ n = 14		0.748^**^ n = 14
	Cell-specific BP (S2, S3, S4)	0.609^*^ n = 14	−0.644^*^ n = 14	0.701^**^ n = 14		0.642^*^ n = 14

*n* is the number of samples; ^**^corresponds to *p* < 0.01, ^*^corresponds to *p* < 0.05 (two-tailed test). Data without stars correspond to *p* > 0.05; “–” means not available for the data set considered.

For the entire water column in the stations excluding S1, BP showed a significant positive correlation with temperature (*p <* 0.01) and DO (*p <* 0.01), and a significant negative correlation with salinity (*p <* 0.01; Table 2). The stepwise multiple linear regression analysis of BP (μmol C m^−3^ h^−1^) with temperature (T), salinity (S), and DO generated the following equation, BP = −1.712 + 0.131 T + 0.017DO (*R*^2^ = 0.682, *p <* 0.01), suggesting that temperature and DO are the most important predictors for BP vertical variation in the whole water columns at S2, S3, and S4. As DOC was only measured at S3 and S4, correlation analysis between BP and DOC could be only conducted at these two stations. BP (*R^2^* = 0.909, *p* < 0.01, n = 11) was significantly positively correlated with DOC in the upper 500 m water column.

We conducted a correlation analysis between BP and environmental factors (temperature, salinity, DO, BA, and chlorophyll-*a*) in the upper 200 m of the water columns at S2, S3, and S4, as well as between BA and cell-specific BP and environmental factors (Table 2). BP, BA, and cell-specific BP were all significantly positively correlated with temperature, DO, and chlorophyll-*a* (*p <* 0.05) but were significantly negatively correlated with salinity (*p <* 0.05). BP was significantly positively correlated with BA (*p <* 0.01). The stepwise multiple linear regression analysis of BP (μmol C m^−3^ h^−1^) with temperature (T), salinity (S), DO, BA, and chlorophyll-*a* was BP = 0.186 + 0.024DO (*R*^2^ = 0.665, *p* < 0.01), suggesting that DO is the most important predictor for BP vertical distribution above 200 m at S2, S3, and S4. BP (*R^2^* = 0.905, *p* < 0.01, n = 8), BA (*R^2^* = 0.867, *p* < 0.01, n = 9), and cell-specific BP (*R^2^* = 0.821, *p* < 0.05, n = 7) were significantly positively correlated with DOC in the upper 200 m water column at S3 and S4.

### 3.4. Bacterial contribution to oxygen loss

**Figure 4:**
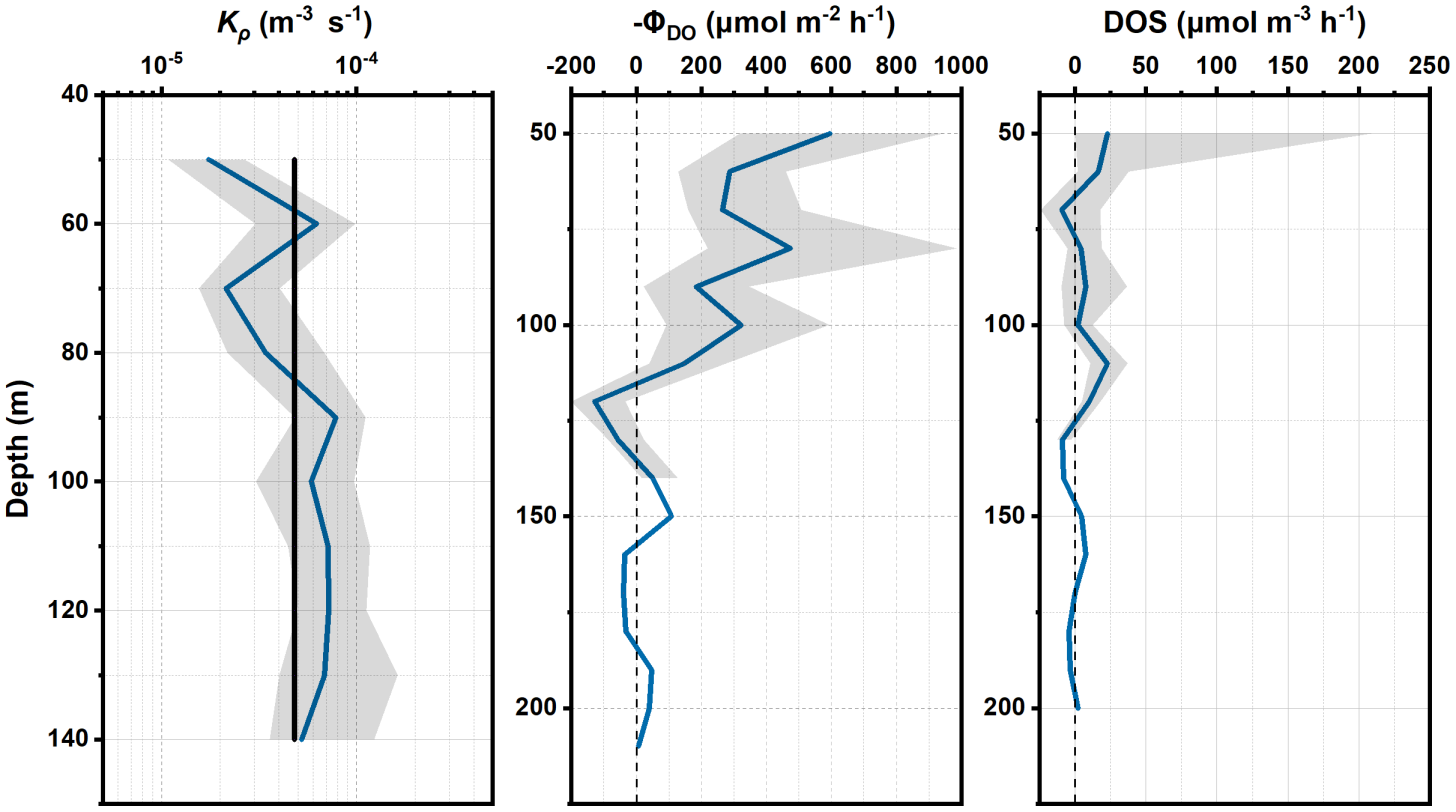
Vertical distribution of *K_ρ_* at depths of 50–140 m with 95% confidence interval (gray shadow) and the constant *K_ρ_* (4.82 × 10^−5^ m^2^ s^−1^; black solid line) that was used for further calculations **(A)**. Vertical distribution of negative diapycnal flux of oxygen ($-\Phi_{\mathrm{DO}}$ ) with depth **(B)**. Vertical distribution of DOS with depth **(C)**.

The *K_ρ_* at depths of 50–140 m at S3 was maintained at the same order of magnitude of 10^−5^, with slight fluctuations (Figure 4A). The average *K_ρ_* (4.82 × 10^−5^ m^2^ s^−1^) at depths of 50–140 m was assumed as a constant for calculations of diapycnal oxygen flux and divergence between 50 m and 200 m.

The diapycnal oxygen flux (DOS) declined with depth between 50 m and 100 m, and stayed stable below 100 m. There were gradient inversions of oxygen at depths of 110–140 m and 160–180 m (Figure 4B).

High DOS values occurred at depths of 50–70 m and 110–130 m. The negative DOS, which corresponded with the positive diapycnal oxygen divergence, occurred at ~70 m and in the range of 130–140 m and 170–180 m, indicating that the layers are the source of oxygen.

The contribution of bacteria to oxygen loss was quantified as the ratio of BOD to average DOS (BOD/DOS) in the upper 50–200 m layers at S3. As shown in Table 3, the maximum average DOS was observed at 50–75 m (10.0 μmol m^−3^ h^−1^). At the depth ranges of 75–100 m and 100–150 m, the average DOS values were only ~45 and 36% of that at 50–75 m, respectively. The average DOS was 1.0 μmol m^−3^ h^−1^ at 150–200 m.

**Table 3:** Estimation of DOS, BOD, and BOD/DOS at depths of 50–200 m at station S3.

Layer	BP	BOD_10%_	BOD_25%_	DOS	BOD_10%_ / DOS	BOD_25%_ / DOS
Oxic Layer (50–75 m)	1.6 (1.3–1.9)	14.5 (12.1–16.9)	4.8 (4.0–5.6)	10.0	145% (121–169%)	48% (40–56%)
Upper oxycline (75–100 m)	1.1 (0.8–1.3)	9.7 (7.4–12.1)	3.2 (2.5–4.0)	4.5	217% (164–269%)	72% (55–90%)
Upper oxycline (100–150 m)	0.7 (0.6–0.8)	6.5 (5.6–7.4)	2.2 (1.9–2.5)	3.6	179% (154–204%)	60% (51–68%)
Core-OMZ (150–200 m)	0.4 (0.2–0.6)	3.6 (1.6–5.6)	1.2 (0.5–1.9)	1.0	378% (172–584%)	126% (57–195%)

The DOS (μmol m^−3^ h^−1^) represents the average negative diapycnal divergence of oxygen at each depth interval. The BCD (μmol m^−3^ h^−1^) and BOD (μmol m^−3^ h^−1^) were calculated from BP (μmol m^−3^ h^−1^) based on BGE of 10 and 25%, respectively. The values in the brackets are the range between the minimum and maximum in the specific layer.

The maximum average BOD appeared at 50–75 m, and the average BOD decayed to ~60, and 25% of the maximum, within the depth range of 75–150 m, and 150–200 m, respectively.

The BOD/DOS values varied along with BGE. The average BOD/DOS ranged from 145 to 378% at a BGE of 10%, but ranged from 48 to 126% at a BGE of 25%. Overall, the average BOD/DOS in the oxic and upper oxycline (120%, 50–150 m) was lower than that in the core-OMZ layer (252%, 150–200 m; Table 3), suggesting that the bacterial contribution to the oxygen loss may be higher in the OMZ. For all depth ranges, the average ratio of BOD to DOS was 153%, indicating that the DOS could not satisfy the overall BOD at the range of 50–200 m.

## 4. Discussion

### 4.1. Comparison of BP in BOB with other tropical oceans

We measured BP at four stations in the southern BOB in January 2020. The BP in the surface in our study was lower than that previously recorded in the western and central BOB during fall; and BP in the upper 150 m in our study was consistent with those in the western and central BOB during winter and fall but was much lower than those during spring and summer (Table 4). Compared with the BP previously recorded in other tropical waters, such as the Arabian Sea (AS), Peninsular Malaysia, Eastern Tropical Atlantic Ocean, and ETSP, BP in the surface or upper 150 m of the BOB across the four seasons was much lower (Table 4). The lack of bioavailable carbon contributed to the low BP in the upper waters of the BOB. In the case of high water temperatures, such as in surface waters, supply of organic carbon is the main factor affecting the distribution of BP (Shiah et al., 2003). High organic carbon supply could stimulate high microbial heterotrophic activity (Shen et al., 2020). The fraction of organic carbon is majorly in the form of DOC than particulate organic carbon (POC; Rao et al., 2021). In our result, DOC showed a significant positive correlation with BP and cell-specific BP at S3 and S4. The DOC concentration in the upper 100 m in ETSP (~50–200 μmol L^−1^; Maßmig et al., 2020) was higher than that observed in our study (~50–85 μmol L^−1^). In the oligotrophic open sea, major labile DOC taken up by bacteria in the upper waters is released by phytoplankton (Lancelot, 1979; Cole et al., 1988; Goldman et al., 1992; Engel et al., 2022). On the one hand, during photosynthesis, phytoplankton produce more carbon than it could be incorporated and release excess DOC into the water; on the other, low molecular weight DOC could be released from phytoplankton cell through passive diffusion (Fogg et al., 1965; Bjørrisen, 1988). Hence, bacterial production is generally related to primary production and chlorophyll-*a* concentration. The mean primary production in ESTP (~35,800 mg C m^−2^d^−1^ in the periods 1980–2005; Pennington et al., 2006) was much higher than that in BOB (~300 mg C m^−2^d^−1^ in the periods 2001–2006; Ramaiah et al., 2010). Similarly, the chlorophyll-*a* concentration was much higher around Peninsular Malaysia (2.06–3.80 μg L^−1^; Lee and Bong, 2008) and ETSP (3.1 ± 1.3 μg L^−1^; Maßmig et al., 2020) than that observed in our study (0.27 ± 0.24 μg L^−1^).

**Table 4:** Bacterial production (μmol C m^−3^d^−1^) data in this and past studies.

Research area		Layer	BP	Sampling time	Reference
Other tropical waters	PM	Surface	340–141,320	2004.09–2005.02	Lee and Bong (2008)
	ETAO	0–150 m	1,395–7,682	1987.07–09	Piontkovski et al. (2003)
	ETSP	0–100 m	0–2,500	2017.04	Maßmig et al. (2020)
India Ocean	AS	Surface	1,312	1994.04–05	Ramaiah et al., 1996
		0–120 m	83–3,333		
		Surface	490	1995.07–08	
		0–120 m	83–1,667		
	WC-BOB	0–120 m	0.8–208	2005.12–2006.01	Ramaiah et al. (2010)
			15–562	2001.07–08	
			0.8–349	2002.08–09	
			29–1,547	2003.04–05	
	WC-BOB	Surface	132	2002.09–10	Fernandes et al. (2008)
		0–120 m	78		
	S-BOB	Surface	50–153 (87)	2020.01	This research
		0–100 m	18–223(88)		
		0–150 m	9–223 (78)		

Studies included those on tropical regions (Peninsular Malaysia [PM], the Eastern Tropical Atlantic Ocean [ETAO], and the Eastern Tropical South Pacific [ETSP]), the Indian Ocean (Arabian Sea [AS] and western and central Bay of Bengal [WC-BOB]), and (southern Bay of Bengal [S-BOB] of this research). Hourly rate was converted to daily rate by multiplying by 24 h d^−1^.

### 4.2. Relative roles of temperature, oxygen, and chlorophyll-*a* on BP

The vertical distribution of BP at S1 was significantly different from that at the other three stations. Therefore, two different kinds of characteristics and controlling factors of BP vertical variation were detected in our study. S1 (nearshore areas) was located off the eastern coast of Sri Lanka and had a more intense OMZ; BP in the OMZ (70–1,300 m) was higher than that in the oxygenated surface (0–70 m), and BP below 1,000 m was much lower and varied slightly. No significant correlation between environmental factors (T, S, and DO) and BP was detected at S1 (Table 2). In contrast, S2, S3, and S4 (open ocean) were in the middle of the southern BOB, where BP was high in the upper 200 m but decreased rapidly below 200 m and varied slightly. The linear model showed that temperature and DO could better predict the variation in BP in the whole water column.

Temperature is found to be the most important factor affecting the vertical variation in BP in the open ocean. From a thermodynamic perspective, proper temperature could facilitate bacterial metabolism by reducing the activation energy required for enzymatic reactions (Feller, 2010). A positive correlation between temperature and BP has been reported in field studies (Puddu et al., 1997; Tuomi et al., 1999). In a simulated culture experiment, BP also decreased with decreasing temperature (Kirchman et al., 1995). A common feature in these studies is that the positive correlation between temperature and bacterial growth was often observed when the temperature is below 20°C (Tuomi et al., 1999; Shiah et al., 2003). In this study, the seawater temperature in the nearly 4,000 m water column had a large span ranging from 2°C to 29°C. Among all environmental parameters, temperature was thus the most important factor controlling bacterial metabolism, including BP.

Regarding DO, its relationship with BP was complicated and depended on the DO concentration range. It is accepted that aerobic bacteria use oxygen as electron acceptors (Cole and Pace, 1995); when DO was decreasing, aerobic bacterial activities would be restricted. For example, Mopper and Kieber (1991) found much slower microbial uptake rates for a suite of small molecules in anoxic waters. In our study, at S2, S3, and S4, the BP and cell-specific BP in non-OMZ layers were significantly higher than those in OMZ layers (Table 1). Additionally, BP showed a rapid, synchronous increase when DO increased from a suboxic condition suddenly. At S1, BP (12.29 μmol C m^−3^ h^−1^) at 200 m (~10 μmol O_2_ kg^−1^) was almost three times as high as that (4.67 μmol C m^−3^ h^−1^) at 150 m (suboxia). Similarly, at S2, BP (0.26 μmol C m^−3^ h^−1^) at 500 m (~15 μmol O_2_ kg^−1^) was almost twice as high as that (0.14 μmol C m^−3^ h^−1^) at 250 m (suboxia). However, a study in the Baltic Sea reported high cell-specific BP at the anoxic–aerobic interface of the water column (Brettar et al., 2012). Cole and Pace (1995) sampled freshwater lakes with different levels of oxygen, and the highest BP was observed just as DO became undetectable. The high BP may be related to special anaerobic and microaerobic bacterial communities. Earlier studies revealed N_2_O concentration maxima around OMZs in many oceanic regions suggested the activity of bacteria and archaea, including BP and respiration, was enhanced in oxygen-deficient environment (Toyoda et al., 2023). Although we did not conduct a simultaneous study on bacterial diversity, Bristow et al. (2017) observed that aerobic communities coexisted with anaerobic communities in the BOB-OMZ. These bacteria could adapt to the hypoxic conditions via variability in carbon respiration pathways to reach more efficient or complete respiration along with the electron tower (Lincy and Manohar, 2020). In anoxic environment, anaerobic microorganisms would use electron acceptors, such as NO_3_^−^, MnO_2_^−^, and SO_4_^2−^, to replace oxygen. For example, heterotrophic denitrifying bacteria would reduce nitrate to nitrite, and further reduce nitrite to ammonia and free nitrogen (Strohm et al., 2007; Lam and Kuypers, 2011), sulfate-reducing bacteria involved in a “cryptic sulfur cycle” could carry out the simultaneous activity of sulfate-reducing and sulfide-oxidizing pathway, as well as sulfide-oxidizing denitrifying bacteria could couple sulfide oxidation to nitrate reduction in OMZ water (Callbeck et al., 2018). The organic matter mineralization mediated by microbial fermentation coupled to sulfate reduction yields ammonium that can drive anammox (Canfield et al., 2010). Therefore, nitrogen-containing organic carbon, such as amino acids, could be utilized more quickly by heterotrophic bacteria in oxygen-deficient and anoxic environments (Van Mooy et al., 2002; Pantoja et al., 2004). In addition, some communities capable of micro-aerobic respiration might have stimulated cell-specific production or the accumulation of particularly active bacterial species (Kalvelage et al., 2011, 2015). However, the oxygen-free condition was not strictly controlled during sampling and incubation in our study. Oxygen in the residual air in the centrifuge tube might result in an increase in DO in the sample, which would have affected BP under suboxia.

Bacterial production may also be closely related to bioavailable carbon. In the oligotrophic waters, labile DOC released by phytoplankton is the main organic carbon source of bacteria in upper waters (Lancelot, 1979; Goldman et al., 1992; Engel et al., 2022). Labile organic carbon is restricted mainly to the upper waters, while most of the organic carbon in the whole ocean is in the form of POC or the refractory high molecular weight DOC (Benner and Amon, 2015; Carlson and Hansell, 2015). POC would dissolve in the mesopelagic waters and provide DOC for bacteria (Cho and Azam, 1988); and could also absorb available DOC as it settles from the upper waters and that DOC could be consumed in the lower layer for bacteria (Hansen and Bendtsen, 2014). In our result, DOC also showed a significant positive correlation with BP in the upper 500 m water column at S3 and S4. Unfortunately, we did not measure DOC and POC in the whole water column; therefore, it was difficult to demonstrate the influence of organic carbon on BP in the whole water column. In addition, the high BP appeared at S1, the nearest nearshore station, might be attributed to the stimulation of bacterial activity resulting from the input of organic matter from the weak upwelling (Supplementary Figure S1), whose supply might reduce the impairment of cell-specific bacterial production under suboxic conditions (Baltar et al., 2009; Maßmig et al., 2020). However, since DOC data was lacking at S1, this speculation needs to be validated by measured data.

Overall, we inferred that there are various major influential factors of BP in different regions. In the open ocean, temperature and DO are the major predictors for BP vertical variation in the water column. Additionally, the extremely high BP in the core of OMZ was observed only at S1, in the layer where DO increased sharply from the suboxia, which might be attributed to the special microaerobic bacterial communities; however, this requires confirmation.

### 4.3. Bacterial contribution to maintaining the BOB-OMZ

Heterotrophic bacterioplankton is the major consumer of oxygen in the ocean, driving the oxygen cycle in the BOB-OMZ and contributing greatly to the formation and maintenance of the OMZ. However, its contribution is difficult to quantify and assess accurately and directly. Maßmig et al. (2020) attempted to compare the BOD and oxygen loss rates to explain the bacterial contribution to oxygen loss. In this study, we referred to their methods to estimate bacterial contribution to oxygen loss at depths of 50–200 m in the southern BOB.

The DOS (231–426 μmol m^−3^ h^−1^; Maßmig et al., 2020) in the ETSP was up to a hundred times higher than that observed in our study (1–10 μmol m^−3^ h^−1^). This huge difference was due to the difference in diapycnal mixing, as there was a considerable spatial variation in diapycnal diffusivities (*K_ρ_*). Maßmig et al. (2020) calculated the DOS in the coastal region off Peru with a high *K_ρ_* (10^−3^ m^2^ s^−1^), as strong turbulence in the coastal upwelling area was prevalent. In our study, the sampling site was in the open ocean and the stratification effect was relatively strong. George et al. (2019) also observed *K_ρ_* (10^−5^ m^2^ s^−1^) below the MLD in the southern BOB, which was the same order of magnitude as our results.

The average estimated BOD at 200 m that we recorded (1.09 ± 0.77 μmol O_2_ m^−3^ h^−1^) was consistent with earlier reports of bacterial respiration rate (0.83 ± 0.17 μmol O_2_ m^−3^ h^−1^) based on measurements of activity of the respiratory electron transport system at approximately 200 m in the BOB (Naqvi et al., 1996). It was expected that the BOD (3–10 μmol O_2_ m^−3^ h^−1^) we measured in the upper oxycline would be lower than that in the ETSP (21–83 μmol O_2_ m^−3^ h^−1^; Maßmig et al., 2020). The Peruvian coastal upwelling in the ETSP is a highly productive marine system, and the chlorophyll-*a* in the ETSP was approximately ten times higher than that in our results (Maßmig et al., 2020). The highly productive environment in the ETSP is suitable for bacterial activity, thus showing a higher BOD than that in the BOB.

In our study, the rapid consumption of DO occurred at the depths of 40–70 m (Figure 2F). The depth for each station corresponded approximately to the end of the euphotic zone and the MLD. The euphotic depth estimated by Secchi disk depth (Walker, 1980) at S1, S2, S3 and near S4 was 62 m, 65 m, 54 m and 46 m, respectively. Correspondingly, the DCM depth at S2 was deeper (75 m) than the other three stations (50 m). Additionally, the MLD occurred at ~60 m at S1 and S2 and at ~40 m at S3 and S4. Therefore, the lack of photosynthetic oxygen input due to light limitation and the lack of atmospheric oxygen input due to physical factors were important mechanisms of OMZ formation in the study region. With DO decreasing, DOS and BOD gradually decreased, and the BOD/DOS increased (Table 3), indicating that the contribution of bacteria to dissolved oxygen consumption gradually increased compared with other oxygen-consuming organisms. In addition, the average ratio of BOD to DOS in the oxic and upper oxycline layers (120%, 50–150 m) was lower than that in the OMZ (252%, 150–200 m), indicating a high potential of bacteria to maintain OMZ. Generally, the average ratio of BOD to DOS in the oxic and upper oxycline layers in our study was 48–217%, which differed from the results (1–62%) estimated using a similar method in the ETSP (Maßmig et al., 2020).

Our estimation of BOD was based on the assumptions about RQ, BGE, and leucine conversion factors, which could vary significantly in the actual measurements. As for RQ, we selected bacterial RQ of 1, which corresponded to complete oxidation of glucose and was commonly applied. However, it could vary from ~0.1 to 4 depending on the substrates used (Rodrigues and Williams, 2001; Williams and del Giorgio, 2005; Berggren et al., 2011). As for BGE, we estimated BGE ranging from 10 to 25% based on the empirical equation from Rivkin and Legendre (2001), which only considers temperature dependence. In fact, temperature could only explain 54% of its high variability (Rivkin and Legendre, 2001), and low-oxygen water may lead to lower BGE (Maßmig et al., 2020). According to another empirical equation [BGE = (0.037 + 0.65BP)/(1.8 + BP)] from Del Giorgio and Cole (1998), the potential BGE ranged from 2.1–2.8% (Supplementary Table S2), which was much lower than that we selected. While the formula is based on extensive freshwater research and is applicable to larger BP values, the BGE calculated using this formula to some extent can reflect that we might have overestimated the actual BGE, and thus the BOD could have been underestimated. Taking the uncertainties of RQ (0.1–4) and BGE (2.1–2.8%) into consideration, the potential BOD could be 1–52 times higher than the maximum BOD estimated in our results. As for leucine conversion factors, we utilized the commonly applied theoretical value of 1.55 kg C mol^−1^ Leu (Simon and Azam, 1989). It was recently found to be varied largely between 0.02 kg C mol^−1^ Leu and 19.20 kg C mol^−1^ Leu in the open ocean based on a large number of published values (Giering and Evans, 2022). However, the range was so huge that the calculated values of BP were lack of comparability. Therefore, despite uncertainties in the leucine conversion factors, based on the best guesses, the average ratio of BOD to DOS was higher than 100%, and could even be underestimated.

Ratios of BOD to DOS higher than 100% indicated either that the diapycnal supply of DO could not satisfy bacterial requirements or that the BP-based conversion factors (e.g., BGE) resulted in BOD that were too high. Assuming that *K_ρ_* did not change noticeably in the whole water column, we estimated the diapycnal oxygen divergence in the lower oxycline, which were 0.02 μmol m^−3^ h^−1^ and 0.01 μmol m^−3^ h^−1^ at 500–750 m and 750–1,000 m, respectively. The diapycnal oxygen divergence below 500 m was much lower than that above 200 m, thus the influence of an oxygen supply from the deep layer to the OMZ could be ignored. In addition, DO could be consumed by other organisms, such as zooplankton and nekton. Giering et al. (2014) estimated that zooplankton could be responsible for ~30% of the total oxygen consumption in the twilight zone of the Northeast Atlantic. There was a huge imbalance in the oxygen budget, suggesting the possibility of other sources of oxygen supply.

We only estimated oxygen supply during diapycnal transport, without considering currents and eddy circulation. For example, the well-ventilated South Atlantic replenishes DO in the suboxic water of the lower thermocline in the North Atlantic (Karstensen et al., 2008). Oxygen-rich waters from AS or the Equator invade the BOB in January, as the eastward Wyrtki jet is blocked by Sumatra and generates a westward pressure gradient (Wyrtki, 1973; Qiu et al., 2009; Nagura and McPhaden, 2010; Wijesekera et al., 2015). Sarma (2002) estimated the oxygen influx of ~15 Tg O_2_ month^−1^ in January between 100 and 1,000 m, while considering horizontal and vertical supply in the southern BOB. We attempted to estimate the bacterial oxygen demand in 1 month in Sarma’s model using the estimated integrated BOD at depths of 100–1,000 m (0.65–1.96 mmol O_2_ m^−2^ h^−1^) and found much higher results (32–96 Tg O_2_ month^−1^) than that of Sarma (2002). This suggests that the horizontal and vertical oxygen supply could still not satisfy the bacterial oxygen consumption in the southern BOB.

Typical mesoscale vortices, including cyclonic and anticyclonic eddies, also exist in the BOB (Chen et al., 2012). Mesoscale vortices play an important part in the transport of water masses and influence biological activities (Chen et al., 2018). Anticyclonic eddies in the BOB could pump DO into the OMZ at any time (Sarma et al., 2016). The sea level anomaly and related surface geostrophic current of BOB in January 2020 showed that S3 was in the anticyclonic eddy during the sampling period (Supplementary Figure S2), which was able to supply indispensable DO for organisms in the upper layers. Sarma and Udaya Bhaskar (2018) reported estimated rates of oxygen input by anticyclonic eddies of 0.39–1.40 mmol O_2_ m^−3^d^−1^ and 0.05–0.10 mmol O_2_ m^−3^d^−1^ at 100 m and between 150 and 300 m during winter in the BOB, respectively. These results were higher than the estimated BOD values (0.06–0.18 mmol O_2_ m^−3^d^−1^ at 100 m, 0.01–0.13 mmol O_2_ m^−3^d^−1^ between 150 and 200 m) obtained in our study, suggesting that anticyclonic eddies could supply a considerable amount of DO.

## 5. Conclusion

Our study revealed the distribution pattern of BP in the southern BOB in January 2020. Bacterial production in the southern BOB was lower than the values reported in other tropical oceans, which is attributed to relatively low primary production and chlorophyll-*a* concentration. In the open ocean, temperature and DO were the main indicators for BP vertical variation in the whole water column. In the nearshore areas, the extremely high BP in the layer with DO sharply increasing from suboxia might be attributed to the special microaerobic bacterial communities.

The average BOD/DOS reached up to 153% at depths of 50–200 m. The imbalance between BOD and diapycnal oxygen replenishment could be reconciled through advection and anticyclonic eddies. Based on our study limitations, we recommend that further measurements of BP and environmental factors in the nearshore areas under suboxia are needed to verify the effect of hypoxia and organic matter. Additionally, simultaneous BOD measurements are needed to compare the estimated values, and more detailed physical-ecological coupling models are required to constrain the oxygen budget of BOB-OMZ in the future.

### Open research

The data used in this manuscript are available from the Science Data Bank (https://www.scidb.cn/s/E7beUz).

## Data availability statement

The datasets presented in this study can be found in online repositories. The names of the repository/repositories and accession number(s) can be found in the article/Supplementary material.

## Author contributions

WY wrote the draft. XM contributed to the data acquisition of temperature, salinity, and dissolved oxygen (DO) concentration measured using CTD. CL contributed to the data acquisition of bacterial abundance. WP, RJ, AW, UW, PDissanayake, GP, and RI provided sampling opportunities in Sri Lanka’s exclusive economic zone and participated the manuscript discussion. RY contributed to the turbulence measurements. YZ contributed to the data acquisition of Chl-a. ZL contributed to the data acquisition of DOC. BW, LR, and JC, participated the manuscript discussion. LS and FZ funded the work. PDu conceived and designed the study. All authors contributed to the article and approved the submitted version.
