# Supplementary material for: Vertical variation of bacterial production and potential role in oxygen loss in the southern Bay of Bengal
Source: Front Microbiol. 2023 Nov 8;14:1250575. doi: 10.3389/fmicb.2023.1250575 (PMC10663246; doi:10.3389/fmicb.2023.1250575)
Supplement: Supplementary file 1 [file Table_1.docx]

Supplementary Material

Vertical Variation of Bacterial Production and Potential Role in Oxygen Loss in the Southern Bay of Bengal

Wenqi Ye, Xiao Ma, Chenggang Liu, Ruijie Ye, W N C Priyadarshani, R M R M Jayathilake, W R W M A P Weerakoon, H B U G M Wimalasiri, P A K N Dissanayake, Gayan Pathirana, R G A Iroshanie, Yuanli Zhu, Zhongqiao Li, Bin Wang, Lu Shou, Lihua Ran, Feng Zhou, Jianfang Chen, Ping Du^*^

*** Correspondence:** Ping Du: [duping@sio.org.cn](mailto:duping@sio.org.cn)

# Contents of this file

Text S1

Figure S1 and S2

Table S1 and S2

# Introduction

The supporting information consists of one text, two figures, and two tables. Text S1 is the supplementary method which showing the detailed calculation of *K_ρ_* and the error calculation of *K_ρ_* and diapycnal oxygen flux and supply. Figure S1 is showing the the sea level anomaly and related surface geostrophic current during our sampling period. Figure S2 is showing the density profiles in the whole water column and upper 200 m water column. Table S1 is showing the sampled depth of parameters in our study. Table S2 is showing the BGE calculated on the relationship of Del Giorgio, P. A., & Cole, J. J. (1998).

# 1 Supplementary method Text S1

# Calculation of Diapycnal Diffusivities (*K_ρ_*)

To estimate the DOS, the CTD sensor (O_2_) was combined with near-simultaneous measurements of the turbulence in the water column at S3. We followed the determination and calculation methods described by Loginova et al. (2019) and Maßmig et al. (2020).

Turbulence measurements were performed using a microstructure profiling system (MSS) from the rear of the vessel. The loosely tethered profiler (MSS90-D, Sea & Sun Technology) was optimized to sink at a rate of 0.55 m s^−1^ and was equipped with three shear sensors and a fast-response temperature recorder, as well as an acceleration sensor, two tilt sensors, and CTD, which sampled with lower response time.

The diapycnal diffusivities (*K_ρ_*, m^2^ s^−1^) were determined at 10 m depth intervals as follows (Osborn, 1980):

$\boldsymbol{K}_{\boldsymbol{\rho}}\boldsymbol{=}\boldsymbol{\Gamma}\frac{\boldsymbol{\varepsilon}}{\boldsymbol{N}^{\boldsymbol{2}}}$

where *N* (s^−1^) is the buoyancy frequency derived from density profiles (Supporting information, Figure S2) over 3 dbar intervals, Γ is the mixing efficiency for which a constant value of 0.2 was used, and ε (m^2^ s^−3^) is the rate of kinetic energy dissipation of turbulence calculated by integrating the shear spectra derived from measurements on a freefalling microstructure probe at S3 (Schafstall et al., 2010).

To reflect the overall diapycnal diffusivities between 50 m and 200 m, a constant *K_ρ_* = 4.82 m^2^ s^-1^, which was the mean *K_ρ_* between 50 m and 140 m, was used to calculate fluxes and divergence of oxygen.

# The error of Kρ and diapycnal oxygen flux

The error estimates of *K_ρ_* is calculated as follows (Schafstall et al., 2010):

$$\boldsymbol{\Delta K}_{\boldsymbol{\rho}}\boldsymbol{=}\boldsymbol{K}_{\boldsymbol{\rho}}\left[ \left( \frac{\boldsymbol{\Delta}\boldsymbol{\Gamma}}{\boldsymbol{\Gamma}} \right)^{\boldsymbol{2}}\boldsymbol{+}\left( \frac{\boldsymbol{\Delta}\boldsymbol{\varepsilon}}{\boldsymbol{\varepsilon}} \right)^{\boldsymbol{2}}\boldsymbol{+}\left( \frac{{\boldsymbol{\Delta}\mathbf{N}}^{\boldsymbol{2}}}{\mathbf{N}^{\boldsymbol{2}}} \right)^{\boldsymbol{2}} \right]^{\frac{\boldsymbol{1}}{\boldsymbol{2}}}$$

And the error of the diapycnal oxygen flux is calculated as follows:

$${\boldsymbol{\Delta}\boldsymbol{\Phi}}_{\boldsymbol{DO}}\boldsymbol{=}\boldsymbol{\Phi}_{\boldsymbol{DO}}\left[ \left( \frac{\boldsymbol{\Delta}\boldsymbol{\Gamma}}{\boldsymbol{\Gamma}} \right)^{\boldsymbol{2}}\boldsymbol{+}\left( \frac{\boldsymbol{\Delta}\boldsymbol{\varepsilon}}{\boldsymbol{\varepsilon}} \right)^{\boldsymbol{2}}\boldsymbol{+}\left( \frac{{\boldsymbol{\Delta}\mathbf{N}}^{\boldsymbol{2}}}{\mathbf{N}^{\boldsymbol{2}}} \right)^{\boldsymbol{2}}\boldsymbol{+}\left( \frac{\boldsymbol{\Delta}\boldsymbol{\nabla}\boldsymbol{C}_{\boldsymbol{DO}}}{\boldsymbol{\nabla}\boldsymbol{C}_{\boldsymbol{DO}}} \right)^{\boldsymbol{2}} \right]^{\frac{\boldsymbol{1}}{\boldsymbol{2}}}$$

Where *K_ρ_* and $\Phi_{DO}$ are the mean profiles calculated from the individual profiles in 10 m depth bins, a constant $\Delta\Gamma=0.04$ is used, the $\Delta\varepsilon$ is the 95%-confidence interval derived by bootstrapping, ${\Delta N}^{2}$ and $\Delta\nabla C_{DO}$ are the standard error of the mean. The standard error here refers to the standard deviation divided by the square root of the number of estimates. The error of diapycnal oxygen supply is derived by error propagation from the flux.

# 2 Supplementary Figures and Tables

# 2.1 Supplementary Figures


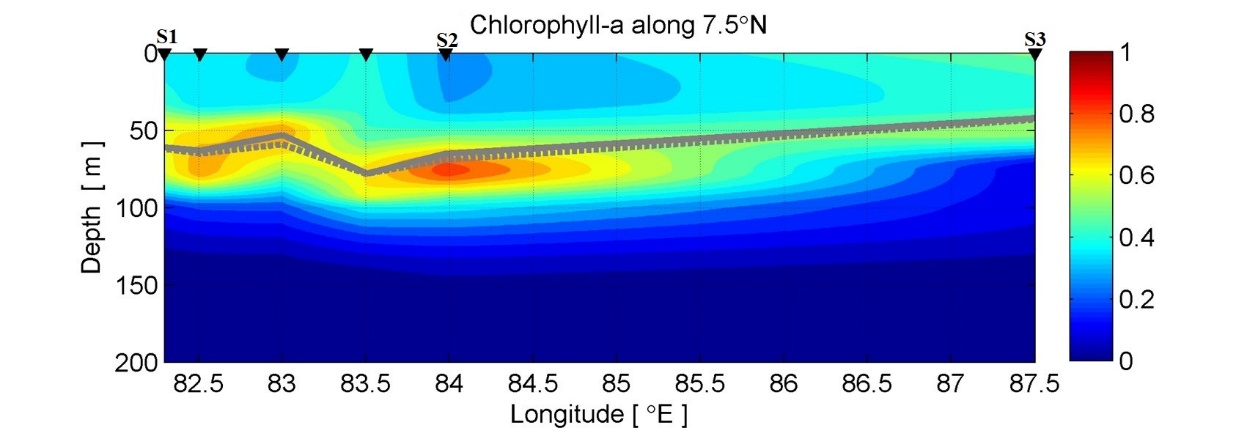


**Figure S1.** Sectional distribution of measured chlorophyll-*a* (mg/m^3^) in the upper 200 m along the 7.5 °N transection during sampling period. Solid and dashed curves indicate mixed layer depth (MLD) and isothermal layer depth (ILD), respectively.


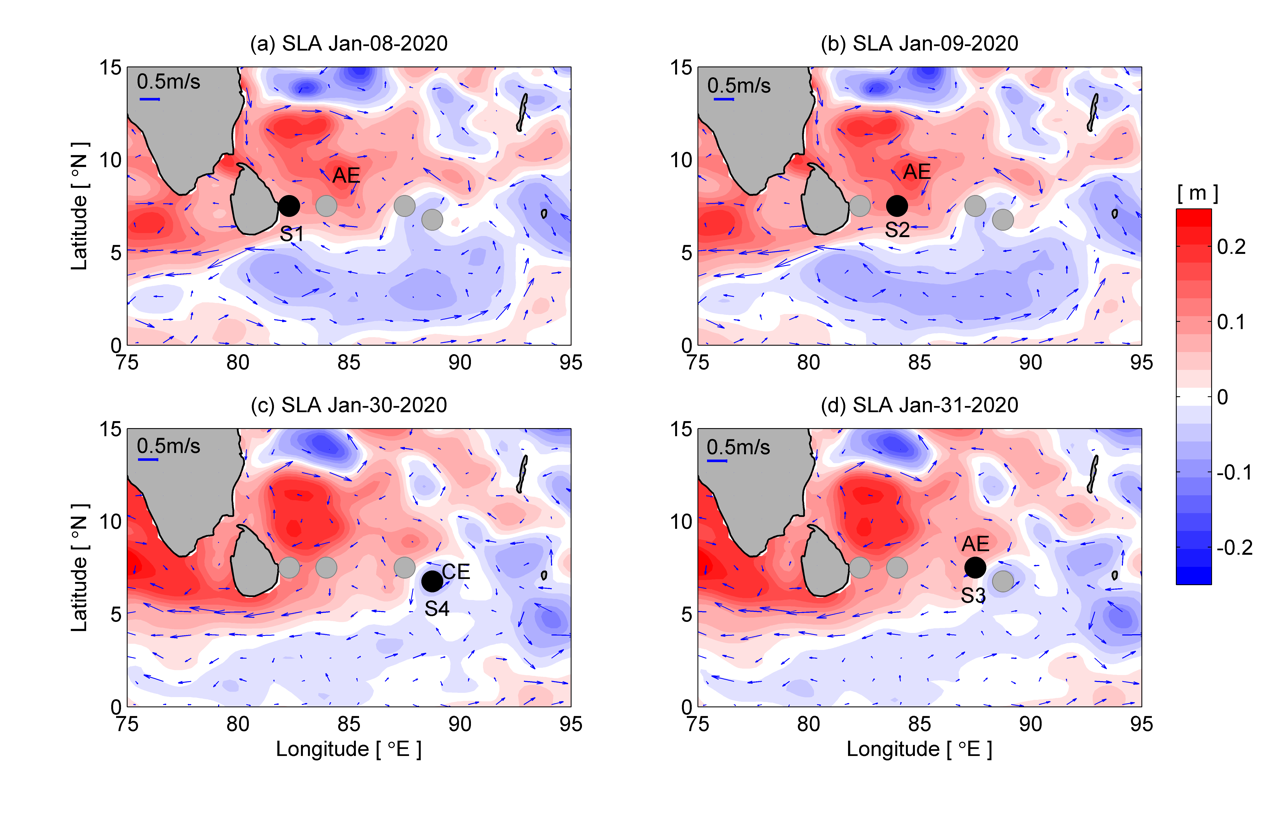


**Figure S2.** The sea level anomaly (shading, with unit of m) and related surface geostrophic current (blue curves, with unit of m s^-1^) in January 2020. Black circles represent four sampling stations. CE: Cyclonic Eddy. AE: Anticyclonic Eddy.

# 2.2 Supplementary Figures

**Table S1.** The information of sampled stations and the sampled depths of chlorophyll-*a*, DOC, BA, and BP

| Station | Longitude (°) | Latitude (°) | Parameter | Sampled Depth (m) | Bottom depth |
| --- | --- | --- | --- | --- | --- |
| S1 | 82.2984 | 7.5011 | Chlorophyll-a | 2, 30, 50, 75, 100, 150, 200 | 3881 |
|  |  |  | BA | 2, 30, 50, 60, 75, 100, 150, 200 |  |
|  |  |  | BP | 2, 50, 75, 100, 150, 200, 500, 1000, 2000, 3800 |  |
| S2 | 83.9781 | 7.5065 | Chlorophyll-a | 2, 30, 50, 75, 100, 150, 200 | 3836 |
|  |  |  | BA | 2, 30, 50, 75, 80, 100, 150, 200 |  |
|  |  |  | BP | 2, 50, 75, 100, 150, 250, 500, 800, 1500, 2000, 3800 |  |
| S3 | 87.5049 | 7.5007 | Chlorophyll-a | 2, 30, 50, 75, 100, 150, 200 | 3730 |
|  |  |  | DOC | 2, 30, 75, 100, 150, 300, 500 |  |
|  |  |  | BA | 2, 30, 50, 75, 100, 150, 200 |  |
|  |  |  | BP | 2, 50, 75, 100, 150, 200, 500, 1000, 2000, 3700 |  |
| S4 | 88.7509 | 6.7596 | Chlorophyll-a | 2, 30, 50, 75, 100, 150, 200 | 3791 |
|  |  |  | DOC | 2, 30, 75, 100, 150, 300, 500 |  |
|  |  |  | BA | 2, 30, 50, 60, 70, 100, 150, 200 |  |
|  |  |  | BP | 2, 40, 75, 100, 150, 300, 500, 1000, 2000, 3700 |  |

**Table S2.** The estimation of BGE based on Del Giorgio & Cole Equation 3

| Depth (m) | BP (μg L^-1^h^-1^) | BGE (based on Equation 3) |
| --- | --- | --- |
| 50 | 0.023 | 2.8% |
| 75 | 0.016 | 2.6% |
| 100 | 0.010 | 2.4% |
| 150 | 0.007 | 2.3% |
| 200 | 0.002 | 2.1% |

*Note:* The Equation BGE=(0.037+0.65BP)/(1.8+BP) is referred to Del Giorgio & Cole (1998) Equation 3.

# Supplementary Reference

Loginova, A.N., Thomsen, S., Dengler, M., Lüdke, J., and Engel, A. (2019). Diapycnal dissolved organic matter supply into the upper Peruvian oxycline. *Biogeosciences* 16(9)**,** 2033-2047. https://doi.org/10.5194/bg-16-2033-2019

Maßmig, M., Lüdke, J., Krahmann, G., and Engel, A. (2020). Bacterial degradation activity in the eastern tropical South Pacific oxygen minimum zone. *Biogeosciences* 17(1)**,** 215-230. https://doi.org/10.5194/bg-17-215-2020

Osborn, T. (1980). Estimates of the local rate of vertical diffusion from dissipation measurements. *Journal of physical oceanography* 10(1)**,** 83-89. https://doi.org/10.1175/1520-0485(1980)010<0083:EOTLRO>2.0.CO;2

Schafstall, J., Dengler, M., Brandt, P., and Bange, H. (2010). Tidal‐induced mixing and diapycnal nutrient fluxes in the Mauritanian upwelling region. *Journal of Geophysical Research: Oceans* 115(C10). https://doi.org/10.1029/2009JC005940
